# Practical Points

**Published:** 1921-09-03

**Authors:** 


### Practical Points.

### MATERIAL FOR FLOORS AND WALLS.

A correspondent asks which is the best material both for floor and walls of an operating-theatre. This is a question about which there still remains considerable diversity of opinion, and it is one to which The Hospital has from time to time devoted much space. In a long series entitled "Hospital Construction: The New Era," published on different dates in 1919, these and similar questions were fully discussed, and we advise our correspondent (F. M. A.) to make a point of reading them.

She urges that terrazzo is not suitable for the floor because it cracks. Terrazzo certainly does crack, but we are not sure, given proper precautions, that it need do so. Rubber flooring is, however, free from this drawback, and is much less tiring to the feet of operators and theatre staff; but it is distinctly expensive.

Lending itself to the formation of floor channels, or hollow skirtings, and the like, terrazzo can easily be cleaned down with a hose, and, being practically cement concrete with a finished face of marble chippings rolled into the cement until it forms a homogeneous mass, it is practically impervious. Of course, the ideally perfect floor would be a jointless floor laid *in situ*, such as the various magnesite floors; but these, unfortunately, are not free from objection on the score of their cracking, although it is only fair to say we have seen some remarkable examples free from cracks. We have our doubts of the durability and particularly imperviousness of such a floor, in which sawdust plays so important a part. The finishing coat of pure material in most of these floors is comparatively thin.

With regard to the wall surfaces, here again the ideal surface would be a jointless one capable of being cleaned down in the same way as the floors, with a hose. Slabs of marble seem to answer this description better than most materials; but glass tiles are, for practical purposes, probably the most suitable. These are set with very fine joints and allow no lodgment for dirt, etc., but tend to become loose and drop after a few years' wear. It should be noted that good results have been obtained by the use of Keene's cement and several of the excellent enamel paints on the market. It would be advisable for the question of wall finishings to be considered conjointly with the question of heating and the maintenance of an equable temperature in the theatre.

